# Selections and Abstracts

**Published:** 1914-02

**Authors:** 


### SELECTIONS AND ABSTRACTS

### PREPARATION FOR THE PROFESSIONS

By Henry S. Pritchett.

The function of education in the modern state is enormous. In one form or another it undertakes to prepare every child for life, every adult for his vocation. Perplexing are the questions which arise in reference to the right method of education for children, not less so are those which have to do with the right training of men for the trades and for the professions.

Back of all questions of educational method in the preparation of men for the professions, lie certain other questions which have to do with the fundamental relations of man in the social order. What is a profession, as distinguished from a trade or a business? What conditions may the state fairly impose concerning the entry of men and women into these professions? To whom shall the state designate the authority and responsibility to enforce the conditions which may be laid down?

Like nearly all other problems of a social or political nature, these problems raise at once an inquiry as to the preservation of the freedom of the individual citizen, and as to the conditions which society may impose upon the individual for its own protection and advancement. The conditions with which society may surround entrance into the so-called professions will vary in the estimation of men according to their conception of the nature of personal freedom.

The passion for personal freedom is perhaps the most striking characteristic of the Teutonic peoples. In the chaos which followed the breaking up af the Roman Empire, the greatest contribution of the Teutonic races lay in their ideals of freedom and loyalty. These peoples chose their own king, but having chosen him, they rendered him absolutely loyalty. These two qualities, freedom and loyalty, are in a large measure supplementary. There is, indeed, no such thing as unlimited freedom. Practical freedom is always limited by duty and loyalty. As Goethe has so admirably expressed it, "He who would have inward freedom must accept outward limitation."

While this conception of freedom was the original heritage of Teutonic peoples, there has been a disposition in our own, and in other nations, to conceive of freedom without the limitations of freedom; to demand the rights of freedom without the responsibilities of freedom; to insist upon the rights of the individual but to deny his responsibility. In social relations, in industrial relations, in political relations, it is one of the problems of our day and of our nation to bring back once more into the thoughts of men the old Teutonic ideal of freedom and loyalty, and to make clear to the individual citizen that only by accepting the limitations of freedom can he enjoy freedom itself.

This ideal is directly involved in the consideration of the conditions which society may demand for admission to the professions. In the State of Indiana, for example, the state constitution directs that the right to practice law shall be guaranteed to every citizen. Does such an enactment make for the freedom of those who desire to enter the profession? On the contrary, the law has been impossible of execution, but if it were carried out, it would make not for the freedom of those who might enter into this profession, but it would impose upon each one of them the necessity to differentiate himself from the mass of the ignorant and the incompetent who have been admitted. The practitioner of the law in the state in which reasonable preparation is asked for admission to the profession, enters into a larger freedom than he who is a member of a profession in a community where no such qualifications are exacted.

In a word, a profession is a calling in which the state confers upon the individual a certain measure of authority and of responsibility; in such a calling society has the right to impose such conditions as shall make for its own protection, and these conditions, if they are reasonably and wisely framed, make also for the greater freedom of him who enters the profession. No man is born with the right to enter a profession in the civilized community any more than he is born with the right to vote. Both of these opportunities are conferred by the state, under such conditions as it may think wise and just, and the establishment of such reasonable tests of admission to these professions makes no less for the freedom of the individual than for the safety of society.

What are the professions, and what conditions may rightly be fixed for admission to them, which shall conserve, on the one hand, individual freedom, and on the other hand, social and industrial efficiency? In the political and social organizations of a hundred years ago, three professions had come to be known distinctly as the learned professions, or more generally, as "the professions," those of theology, of law, and of medicine.

The qualities which distinguished these callings from those of trade and business rested essentially upon two grounds: First, for the practice of a profession it was assumed that a high order of education and a fair amount of special training was necessary. They were, in other words, expert callings. In the second place, these callings imposed upon the individual a certain quasi-public relationship. It meant something more to be a preacher or a lawyer or a physician than to be a merchant or a tradesman. It meant that the community conferred upon the individual a certain public recognition, and that the individual, in virtue of that recognition, accepted a certain public responsibility. The professons were, therefore, not callings in which the individual could consider his own interests alone. He must, from the very nature of the calling, consider the interests of the public as well, and by virtue of the recognition which the public afforded him, he accepted a certain obligation to deal fairly by the whole community and in the light of public interest. The idea is well expressed in Bacon's famous phrase, "I hold every man a debtor to his profession." In other words, a profession is a calling in which by reason of its public relationship he who enters it, whether he formally agrees to do so or not, owes to his profession the duty to regard the public interest as well as his own interest. These two characteristics, the variety and extent of the technical preparation, and the nature of the public relationship which is conferred, serve to set aside the professions from those ordinary callings by which men earn their livelihood.

The list of professions is today greatly enlarged. It includes not only the preacher, the lawyer, the physician, but the teacher, the engineer, the architect, and the various technical callings involving applied science. It is true that these professions do not have equal responsibilities or equal advantages. They do not receive at the hands of the state equal recognition and authority. But they all share, in a greater or less degree, in the two fundamental characteristics which differentiate a profession from a business.

If one grants that society has the right to impose conditions upon the entry into these professions, that such conditions make for a larger freedom of the individual and for the larger service of society, one faces naturally the inquiry, "What are the conditions which society should impose in order to secure at once its own interest and to promote the happiness and freedom and usefulness of those who enter the true professions?"

The fixing of such conditions has proved in all countries matters of difficulty. There are under modern conditions an army of ill-prepared and unfit men, anxious to enter these professions, desirous of obtaining not only the emoluments which may possibly be had from their practice, but also to share in whatever credit may attach to membership in a recognized profession. Such men desire admission upon the easiest terms. They are ready at all times to invoke the plea for individual freedom, and to insist that the state make such conditions as will allow the freest entry into any profession. Against this tendency, prompted in part by selfish interest, in part by ignorance, he who stands ready for the interests of the whole race must be ready to do battle: Not only must he who stands for such public service oppose the ordinary arguments, but he must expect to be assailed at every step, upon the ground of the limitation of that universal freedom into which all civilized men claim to be born. A part of the duty of him who strives for a high order of professional education is to transform the popular conception of what freedom in a democracy means.

In the framing of such conditions as the state may impose in the interest of the great body of citizens, the endeavor should be to make such conditions as may be uniformly fair and just: conditions which bear equally on all parties or sects or associations of men. A large part of the difficulty of enforcing such conditions will be overcome if they can be so justly framed as to command the respect of impartial judges.

In the second place, a very fair discrimination should be made between those callings which are true professions, which demand a high order of education, high qualities of originality and resourcefulness, and which involve a true public responsibility, and certain other callings allied or related to them, which however have totally different responsibilities and demand totally different qualities.

For example, the profession of medicine calls for qualities of the highest order. To be a physician in the best sense one must be an educated man. He must be of sound judgment; he must be resourceful; he must recognize his duty as a public servant. The vocation of pharmacy is closely associated with medicine. There has been a dispotition in many quarters to demand for those who enter this calling qualifications equal to those which are asked for admission to the practice of medicine. And yet the qualities called for are essentially different. The pharmacist is not asked to have great initiative, to take great responsibilities, to deal with large public interests. The qualities which are demanded of him are accurate technical preparation within a narrow range, fidelity and care. To demand of him who wishes to become a pharmacist the same qualifications, and to impose in his preparation the same conditions which are demanded of him who wishes to practice medicine is clearly unjust. The one is a profession; the other is not. And even among vocations which are rightly named professions it still remains true that the exact conditions for admission may well show careful and just discrimination.

Admitting the general attitude toward the whole question on the part of those who represent the state, which has been expressed in these general statements, the conditions which may be insisted on as prerequisites to the practice of a profession seem to me to be embraced under two general heads: First, the requirement of a good, general education. Second, a thorough grounding in the fundamental, underlying sciences and technical branches of knowledge upon which the profession rests. When requirements are broad, they make no distinctions as between sectarian bodies; they are as simple as society can afford to accept.

The requirement of a good, general education is perhaps, on the whole, the greatest safeguard which a commonwealth may set up as a protection for itself, at the doorway of the great professions. The experience of other nations shows that no specific and detailed set-up requirements can insure those qualities of mind and heart which are called for in the exercise of any of these great callings. However necessary it may be that he who practice medicine may know anatomy, that he who practices engineering shall know physics, that he who practices law shall know pleading, it still remains true that these things furnish no test of the texture of the man's soul. As to this there is no test at once specific and final and exact, but the experience of all civilized nations goes to show that the possession of a broad, general education is the surest means to the symmetrical development of those powers of mind and heart which go to make up a true man. Whatever may be the technical skill of him who is to enter a profession, it is first of all to be desired that he shall be a man. There is no specific in education for making a man. The best we can do is to ask that he shall at least have passed some years in touch with great minds and noble thoughts and high ideals, and this is compassed so far as human conditions can bring about such a result, by the requirement of a good general education.

Of course, the practical question arises, "What is a good, general education?" It is being variously answered in different states of this nation by demanding for candidates to the great professions the completion of a high school course or of two years in college, or in some cases by the completion of a full college course. With regard to the practical determination of this matter it may be said that in some states the enforcement of a good four-year high school course, before taking up the study of law, or of medicine, or engineering, is a higher standard than is the requirement of two years of college work in other states. Common sense will dictate that in the enforcement of such conditions, standards shall not be elevated beyond what is possible; that the reform of the whole educational system can not be made in a day; that it is better to demand and enforce a reasonable standard than to demand and fail to enforce an impossible standard.

In the various states of our Union illustrations of all phases of this situation can be met. There are states today where the enforcement of even a four-year high school course, as a preliminary for the study of law or medicine, would be a very high standard. In most states, however, this would be a very low standard. In the long run, we shall need to consider both the character of the general education which the candidate is to receive, and the length of time which is required to complete it. We can not afford to send into the professions ill-prepared youths, nor yet can we afford to keep them so long in the schools that they lose somewhat of that resiliency which a man ought to carry into a profession. It may well be hoped that as secondary school and college improve, the intellectual stage which a man reaches at the end of two years of college shall be higher than that which he has hitherto attained. It ought certainly to be possible to establish an educational system which shall fit men to begin their professional studies by the time they are twenty-one years of age. In the confusion which exists today among our various states, it will be necessary for each state to aim at the highest standard for professional entrance consistent with its educational system. It is a matter full of hopefulness to remember that very rapidly our states are approaching a uniform excellence in their secondary school systems, and not many years will elapse before secondary education in one part of the country will be upon the same plane as secondary education in every other part. But until that time comes each state must translate the requirement of a general education in accordance with the facilities and opportunities which the school system of the state affords, and have the courage to raise these standards as rapidly as conditions improve.

The enforcement of adequate grounding in the technical subjects which underlie the great professions seems so evidently necessary as to require no argument, and yet today a very large proportion, perhaps the majority, of those who undertake the two professions most directly touching the life of the nation—the practice of medicine and the practice of law—are not required to make such preparation and do not make it.

The matter can be illustrated best by a brief reference to the existing conditions of admission to the practice of medicine. We have in our country an extraordinary, though now decreasing, number of medical sects. In no other country do they flourish to the same extent for the reason that in no other country is the way made so easy for them to enter the profession of medicine without adequate training. Now, modern medicine is nonsectarian. It is scientific. It turns exactly the same fact to the osteopath, the homeopath, the Christian Science healer, the naturepath and to all other sects. Scientific medicine simply undertakes to deal with the observed phenomena of the human body without prejudice. It will gladly accept a remedy of the homeopath if it has been proved to have value. It will gladly use the services of an osteopath in those cases in which its value has been shown. It will intelligently seek to use the mind itself in the treatment of disease along reasonable scientific lines. But whatever form of medicine one practises, whether he take up scientific medicine without allying himself to any sect, or whether he chooses to ally himself with one of the medical sects, it still remains true that he must deal with the same human body and its diseases. Whether he call himself a homeopath or an osteopath or a Christian Science healer, or simply a physician, he still has the same need to understand the physiology, the anatomy and the chemistry of the human body.

It is no part of the state's business to say that a man shall not be a homeopath or an osteopath or a Christian Science healer, but the state does have the right and the duty to say that whether a man practice under one name or another, he shall be thoroughly grounded in the knowledge of the anatomy and physiology of the human body, and that he shall know those biological and chemical sciences upon which such knowledge depends. This requirement is absolutely fair. It makes no discrimination between the various medical sects. It is the least protection which society can afford to accept, and it is the minimum requirement which any state ought to make of those who wish to enter into the practice of medicine, whether they desire to practice under one name or another.

Exactly the same may be said of the practice of law. There do not exist in the law, as in medicine, legal sects. It is none the less true that no man is ready to go into the practice of law who will not give a fair study to the underlying subjects upon which the study of the law rests.

It therefore seems clear to me, in the light of all experience of civilized nations, that the requirements for entry into the great professions shall be: first, the acquirement of a general education which shall guarantee at least some contact with the best thought and the best ideals of civilization, and second, so thoroughgoing a preparation in the subjects which underlie the practice of the profession as to give the practitioner a real ability to judge. No conditions can be devised which will be sufficient to keep out all the unworthy, but these two simple conditions which are fair and just, which do not abridge the liberty of the individual and which conserve the public interest, will at least give us any state of the Union a body of practitioners who can think and deal intelligently with the phenomena which they are called upon to handle.

There remains yet one other practical question to be settled in the discussion of such a subject, and that is to determine, in any given commonwealth, the hands in which the enforcement of such conditions shall lie. Who shall represent the state in determining the conditions of admission to the professions? Who shall be charged by the state with the enforcement of these conditions?

In most states of our Union this question has been met in the most effective way. Each state has, to be sure, a department of education, but these departments have been clothed, as a rule, with limited powers and responsibility. The questions of the conditions of admission to the professions have been entrusted in most cases to other boards not in touch with the schools of the state or its educational conditions. The question of fitness for a profession is preeminently an educational question and the administration of this matter would naturally be entrusted to a department of education in touch with the schools and in sympathy with their ideals both of freedom and of discipline.

This great state, the most populous of all our states, is to be congratulated upon having instituted a Department of Education upon which has been conferred a larger power and a larger responsibility than most states have been willing to consider. It is in honor of this great agency of education that we here meet today. The power which it has received in the past has contributed enormously to the upbuilding not only of the general school system of the state, but to the improvement of the professions as well. We may well believe and hope that as times goes on, this agency, with still greater powers, with still larger responsibilities, will go forward to develop those standards of general and professional education which make alike for the highest freedom of the individual citizen, and for the truest progress of the whole commonwealth.—Quarterly of the Federation of State Medical Boards.

---

### STAFF OF GRADY HOSPITAL.

At a recent meeting of the trustees of Grady Hospital, the following officers and staff were elected:

Joseph Hirsch was re-elected president, Col. Robert J. Lowry was elected vice-president to succeed the late Dr. George Tigner, and Wade P. Harding was elected secretary.

Mr. Hirsch has been president of the board since the establishment of the hospital, twenty-two years ago.

The entire medical board was re-elected, as follows:

L. B. Clarke, M. D., president.

C. W. Strickler, M. D., vice-president.

L. P. Stephens, M. D. secretary.

Department of Medicine.—R. T. Dorsey, M. D.; assistant, W. E. Quillian, M. D. C. W. Strickler, M. D.; assistant, J. E. Paulin, M. D. J. C. Olmsted, M. D.; assistant, J. H. Hines, M. D. L. B. Clarke, M. D.; assistant, W. A. Upchurch, M. D. L. P. Stephens, M. D.; assistant, J. N. Brawne, M. D. G. Pope Huguley, M. D.; assistant, T. C. Hodge, M. D.

Department of Surgery.—James N. Ellis, M. D.; assistant, L. T. Pattillo, M. D. W. S. Goldsmith, M. D.; assistant, W. E. Persons, M. D. Willis Jones, M. D.; assistant, F. G. Hodgson, M. D. F. K. Boland, M. D.; assistant, J. O. Kinard, M. D. E. G. Jones, M. D.; assistant, W. E. Yankey, M. D. W. F. Westmoreland, M. D.; assistant, T. C. Davison, M. D.

Department of Gynecology and Obstetrics.—George H. Noble, M. D.; assistant, O. T. White, M. D. W. A. Crowe, M. D.; assistant, L. Sage Hardin, M. D. S. T. Barnett, M. D.; assistant, John F. Denton, M. D.

Department of Eye, Ear, Nose and Throat.—F. P. Calhoun, M. D.; assistant, Thomas H. Smith, M. D. R. B. Ridley, Jr., M. D.; assistant, none. John D. Thompson, M. D.; assistant, none.

Department of Pathology.—Claude A. Smith, M. D.; assistant, none. E. C. Thrash, M. D.; assistant, A. H. Bruce, M. D. John Funke, M. D.; assistant, none.

Dr. F. G. Hodgson was elected as junior orthopedic surgeon, assistant to Dr. Michæl Hoke.

